# Evaluating large language models on multilingual vaccine knowledge: a benchmark study

**DOI:** 10.1038/s41541-026-01500-1

**Published:** 2026-06-10

**Authors:** Siyuan Chen, Lily Wass, Zhengdong Wu, Lucas Garay, José Vizoso, Kathy Leung, Joseph Wu, Leesa Lin

**Affiliations:** 1 Laboratory of Data Discovery for Health Limited (D24H), Hong Kong Science Park, Hong Kong SAR, China; 2 Moonrise Initiative, Hong Kong SAR, China; 3 WHO Collaborating Centre for Infectious Disease Epidemiology and Control, School of Public Health, Li Ka Shing Faculty of Medicine, The University of Hong Kong, Hong Kong SAR, China; 4 Division of Emerging Interdisciplinary Areas, The Hong Kong University of Science and Technology, Hong Kong SAR, China; 5 The University of Hong Kong - Shenzhen Hospital, Shenzhen, China; 6 Department of Infectious Disease Epidemiology & Dynamics, London School of Hygiene and Tropical Medicine, London, UK

**Keywords:** Computational biology and bioinformatics, Diseases, Health care, Medical research

## Abstract

Large language models (LLMs) are increasingly used by clinicians and the public for vaccine information, yet their factual accuracy across languages and vaccine domains remains insufficiently characterized. We evaluated 13 LLMs using VaxEval, a multilingual vaccine-knowledge benchmark of 1886 vaccine-related multiple-choice questions spanning 14 vaccines in English (71%), Spanish (13%), and Chinese (16%). All items underwent quality control, with reference answers verified against authoritative guidance and peer-reviewed sources. Model performance was evaluated under zero-shot, few-shot, and chain-of-thought (CoT) prompting, with exact-match accuracy defined as selecting the pre-specified reference option. We used mixed-effect logistic regression to estimate associations between model group (newer flagship models vs earlier models), prompting strategy, language, and vaccine type, and answer correctness. Mean accuracy across models was 86.0% in English, 83.7% in Spanish, and 80.0% in Chinese. Flagship models had higher odds of correctness than earlier versions (OR 1.57; 95% CI 1.50–1.65; *P* < .001). Few-shot prompting was associated with higher correctness (OR 1.17; *P* < .001), whereas CoT prompting was associated with lower correctness (OR 0.79; *P* < .001). Performance varied by vaccine type and question category, underscoring the need for rigorous evaluation, structured guardrails, and targeted refinement before using LLMs for vaccine communication.

## Introduction

Large language models (LLMs) have rapidly advanced and are increasingly used for knowledge-intensive tasks, including health information seeking. However, strong performance on general medical examinations does not necessarily translate to reliable, safe use in public-facing contexts, where errors may be persuasive, difficult for lay users to detect, and potentially consequential. Vaccine communication is a particularly high-stakes domain, given the need for guideline-sensitive, jurisdiction-specific recommendations and the societal consequences of misinformation and declining confidence. Accordingly, this study aims to develop a multilingual vaccine-knowledge benchmark (VaxEval) and systematically evaluate the factual accuracy of widely used LLMs on vaccine-related questions.

Recent models such as GPT-4, GPT-4o, Claude, Gemini, and others have demonstrated strong performance on standardized medical question-answering tasks, including multiple-choice examinations in specialized domains^1–7^. These results suggest that LLMs can capture substantial medical knowledge and may support clinical and educational workflows. Nevertheless, exam-style accuracy often reflects recognition of canonical facts under controlled conditions and may not capture real-world deployment risks such as overconfident errors, failure to follow clinical guidelines, or sensitivity to prompt framing and dataset composition. These limitations are particularly salient for vaccine communication, where small deviations from guideline-concordant advice (e.g., dosing intervals, contraindications, or eligibility) can translate into actionable decisions and may be difficult for lay users to detect or correct. Rigorous, domain-specific evaluation is therefore a prerequisite for responsible use.

In practice, LLMs are already being explored for health communication, literature synthesis, and operational support in clinical and research settings^8–11^. At the same time, reliable use requires rigorous validation across domains and modalities of questioning. LLM outputs may be inconsistent, may omit key contraindications or dosing constraints, and may generate plausible but incorrect rationales—failures that are especially problematic when users treat outputs as authoritative. These concerns motivate careful benchmarking that evaluates not only average accuracy but also heterogeneity across topics, populations, and languages.

Evaluating vaccine-related knowledge is urgent. Vaccine hesitancy remains a critical global health challenge, recognized by the World Health Organization as a major threat^12^. Corrective and clearly contextualized vaccine information can improve confidence and uptake, whereas inaccurate or oversimplified guidance may reinforce misperceptions or lead to inappropriate decisions. Population surveys indicate rapidly rising adoption of LLM chatbots, and health-focused polling suggests that a subset of adults already use AI chatbots to seek health information—often with limited confidence in judging accuracy—raising the stakes for guideline-sensitive topics such as vaccination^13,14^. This underscores the need to understand when these systems succeed, where they fail, and whether performance differs across languages and vaccine domains. Although LLMs may help disseminate accurate vaccine information, they also risk producing errors that could exacerbate hesitancy or undermine trust^15,16^. Additionally, as the model ecosystem expands, comparative evaluation across commonly accessible systems becomes essential for evidence-informed deployment choices.

To address these gaps, we developed VaxEval, a multilingual vaccine knowledge benchmark spanning 14 vaccine types and three UN languages (English, Spanish, and Chinese). The benchmark comprises quality-controlled multiple-choice questions with reference answers verified against authoritative global health guidance and peer-reviewed literature. Using VaxEval, we systematically evaluated 13 widely used LLMs under standardized prompting protocols and quantified determinants of response accuracy across model generations, languages, vaccine domains, and question categories.

## Results

### Dataset characteristics and descriptive overview

The final dataset comprises 1886 vaccine-related MCQs across three UN languages: English (71%, 1340), Spanish (13%, 250), and Chinese (16%, 296). English items were drawn from global health authorities (918) and peer-reviewed journals (422), thereby combining guideline-oriented questions with research-facing, potentially more heterogeneous content. Overall, the benchmark spans 14 vaccine types and captures substantial variation in topical emphasis across languages and sources, providing a broad and challenging testbed for multilingual LLM evaluation.

In terms of vaccine representation, HPV was the most represented vaccine (276 MCQs; English 212, Spanish 25, Chinese 39), followed by COVID-19 (230; English 145, Spanish 23, Chinese 62) and influenza (192; English 138, Spanish 21, Chinese 33). The Spanish subset included relatively more items on shingles (24) and RSV (22), whereas the Chinese subset emphasized COVID-19 (62) and influenza (33) and contained fewer RSV-related questions (2). Detailed distributions by vaccine type and source are provided in Supplementary Fig. 3.

**Fig. 1:**
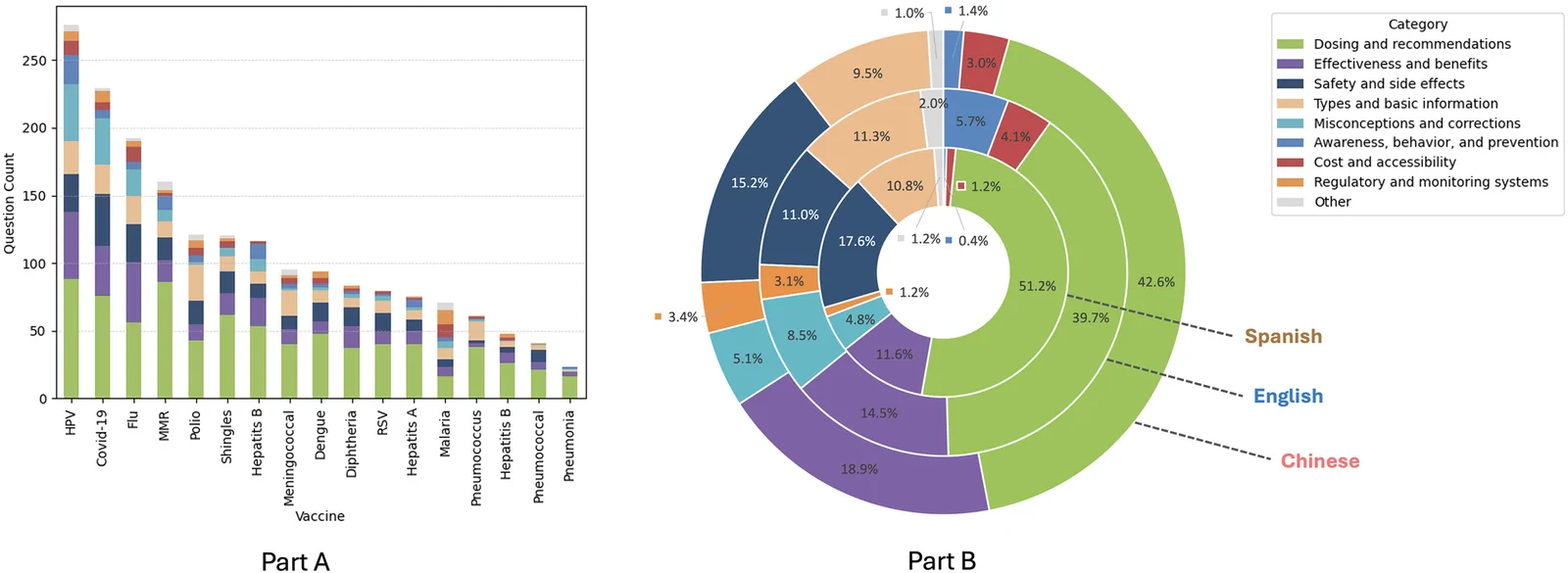
Distribution of vaccine-related question categories by language and vaccine type. **A** Breakdown of question categories by vaccine; **B** breakdown of question categories by language.

Across the full dataset, questions most frequently concerned dosing and recommendations (41.73%), such as “Is it necessary to get vaccinated for COVID-19 if you already have antibodies?”, which underscores the importance of clear guidance on vaccination schedules and administration practices (Fig. 1A). Items on effectiveness and benefits (14.85%), safety and side effects (12.51%), and types and basic information (10.98%) further highlight the public’s demand for clear evidence on vaccine efficacy, safety profiles, and formulations available. These categories are especially relevant for vaccines like COVID-19, where rapid development and deployment have heightened public scrutiny. The category of misconceptions and corrections (7.42%) signals the persistent challenge of misinformation, emphasizing the role of public health authorities in delivering accurate, targeted messaging to diverse audiences. Awareness, behavior, and prevention (4.35%) focused on the importance of protective health behaviors like vaccination to mitigate vaccine-preventable diseases, while cost and accessibility (3.55%) contained questions related to accessing vaccinations, such as through national immunization programs. Finally, regulatory and monitoring systems pertain to the methods of vaccine safety surveillance and quality control (2.86%). As shown in Fig. 1B, the distribution of vaccine-related question categories is broadly comparable across the three languages. “Dosing and recommendations” represents the most prevalent category in each language— English (39.7%), Spanish (51.2%), and Chinese (42.6%).

**Fig. 2:**
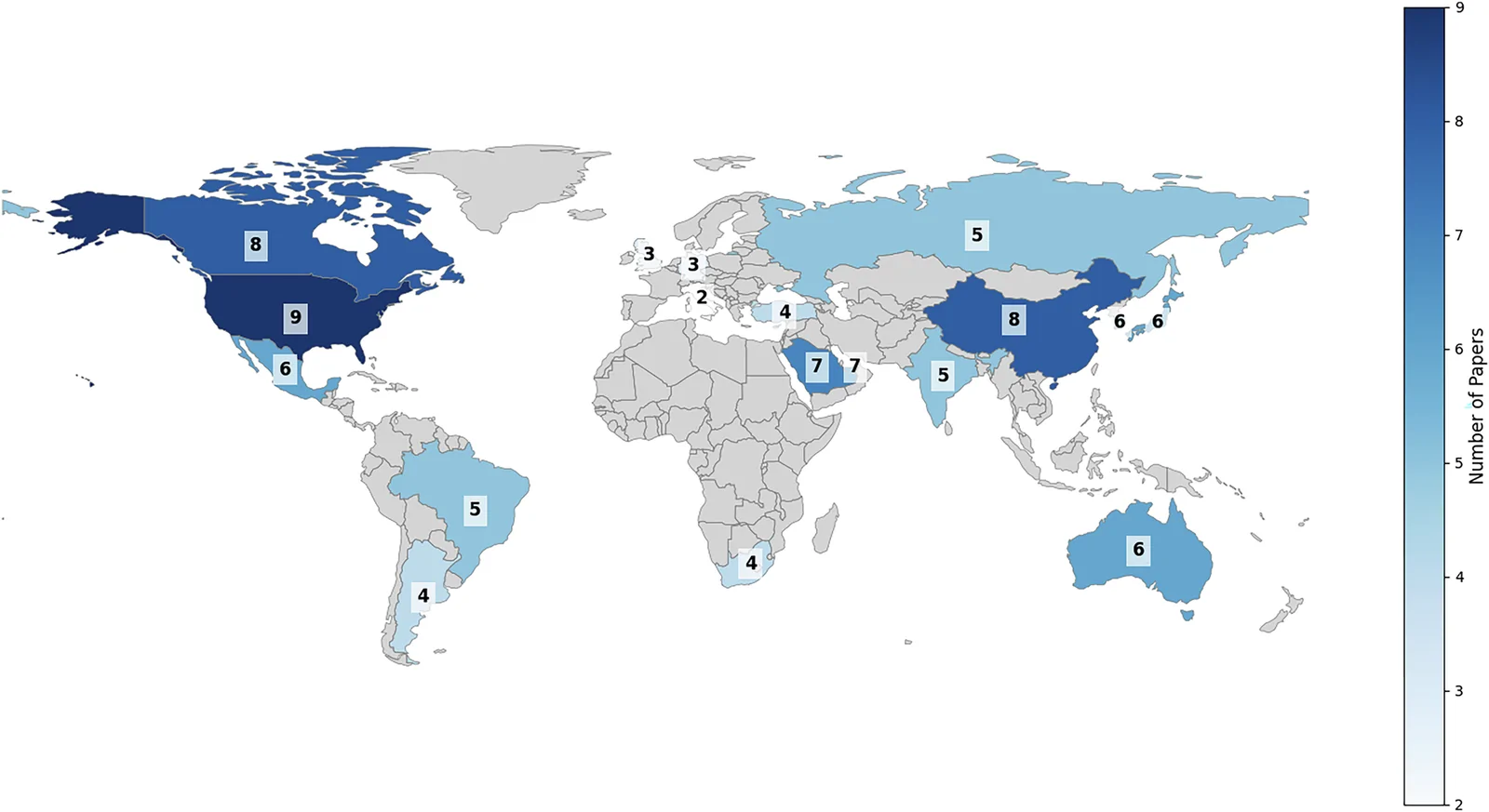
The number of peer-reviewed articles sourced from each country in the English Source Two dataset.

Finally, the peer-reviewed English Source Two subset illustrates geographical disparities in research coverage (Fig. 2). Research from the United States and Canada covered 7–9 of the 14 vaccine types, China addressed around six types, and Europe demonstrated moderate diversity (5–6 types), whereas countries in Africa and South America typically focused on 1–2 types. Target-population distributions within the peer-reviewed subset are summarized in Supplementary Fig. 4. Collectively, these characteristics underscore that VaxEval reflects not only multilingual variation but also substantive heterogeneity in vaccine focus, question type, and evidence source—context that is essential for interpreting downstream performance differences reported below.

### Overall model performance and main effects

Table 1 shows the performance of 13 LLMs in answering MCQs on 14 different vaccines using three prompt types (Zero Shot, Few Shot, and chain-of-thought (CoT)) across three languages. For the complete table, please refer to Supplementary Table 1. Each cell in the table indicates the percentage accuracy achieved by a model for a specific prompt type and language.

**Table 1:** Comparison of overall accuracy (%) across large language models for different languages and evaluation settings

Model	English	Spanish	Chinese	Overall
Flagship				
GPT-4.5	89.30%	76.00%	88.4%^a^	87.40%
Llama-4-Maverick	89.6%^a^	94.4%^a^	88.9%^a^	90.20%
Deepseek-v3	89.10%	91.7%^a^	89.9%^a^	89.60%
Grok-3	90.1%^a^	81.30%	71.70%	86.30%
Qwen2.5	86.30%	78.50%	86.50%	85.30%
GLM-4	82.10%	78.00%	83.50%	81.80%
Earlier				
GPT-3.5-Turbo	77.70%	79.60%	77.40%	77.90%
GPT-4	86.90%	89.20%	76.40%	85.70%
GPT-4o	90.5%^a^	92.7%^a^	87.05%	90.30%
Gemini-1.5-Pro	88.40%	81.60%	45.50%	81.30%
Claude-3-Opus	85.00%	77.50%	88.70%	84.50%
Yi-Lightning	80.10%	79.30%	74.30%	79.20%
Reka-Core	83.50%	88.00%	82.00%	83.90%
Methods				
Zero shot	85.00%	90.30%	79.90%	84.80%
Few shot	86.90%	90.20%	81.80%	86.30%
CoT^b^	86.10%	70.60%	78.20%	77.80%
Overall	86.00%	83.70%	80.00%	

^a^Underlined accuracy values denote the top three performing LLMs for each language on vaccine-related questions.

^b^*CoT* chain of thought.

### Performance heterogeneity across vaccines and question categories

**Fig. 3:**
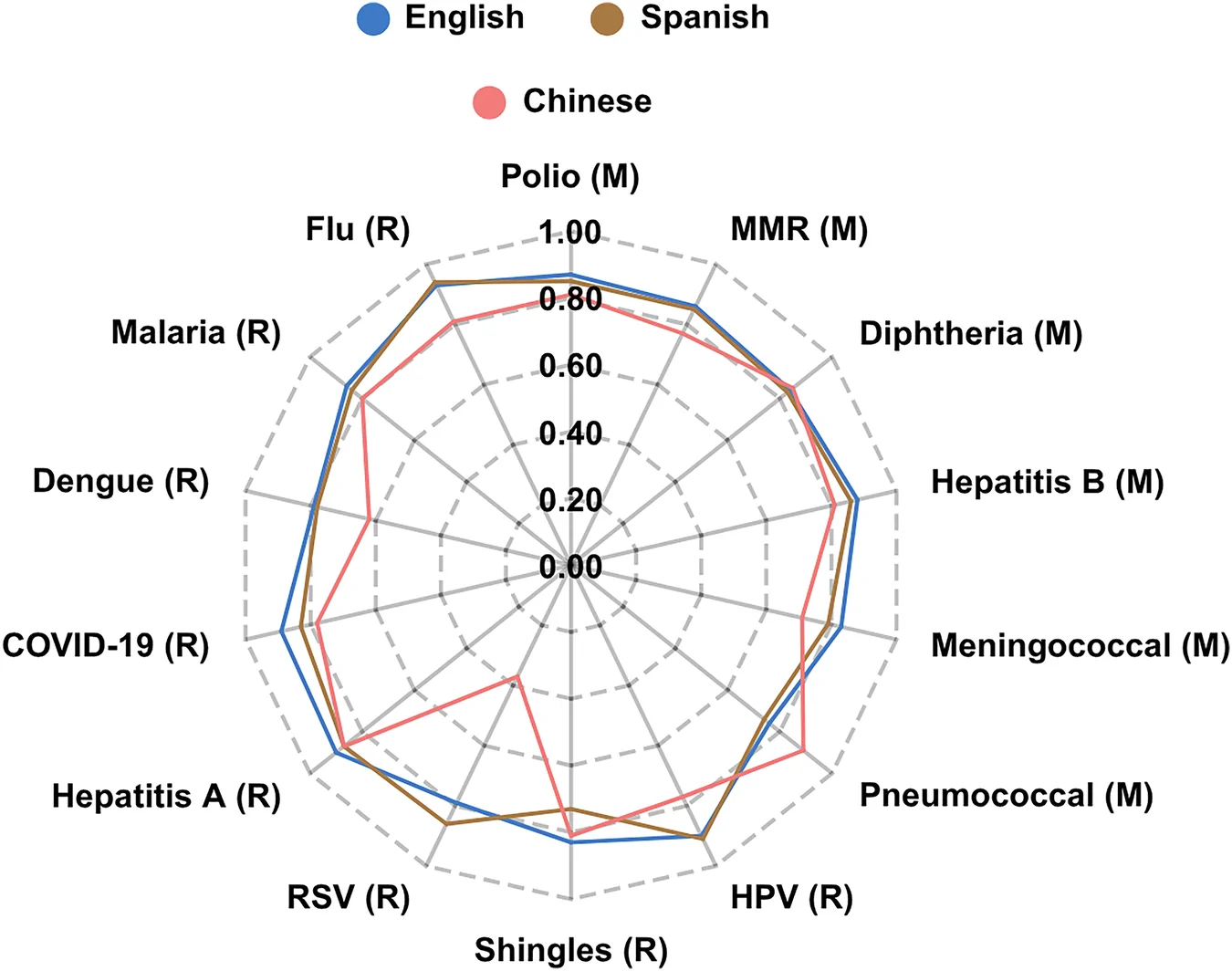
Radar chart of LLM accuracy on 14 vaccines across three languages. M mandatory vaccine schedule, R recommended.

Figure 3 illustrates the variation in accuracy across different LLMs when answering questions about each vaccine type in three languages. On average, the LLMs achieve an accuracy of 82.1% for mandatory vaccines and 81.0% for recommended vaccines. Mandatory vaccines, Meningococcal (77.3%) and Pneumococcal (79.7%) vaccines, show lower performance. In contrast, performance accuracy was higher for vaccines such as Polio (84.3%), MMR (82.5%), Diphtheria (84.2%), and Hepatitis B (84.8%). Among the recommended vaccines, LLMs generally perform well for more common vaccines, such as Hepatitis A (88.0%), HPV (85.9%), and Influenza (89.3%). However, for less common vaccines like Dengue (72.7%), RSV (67.4%), and Shingles (78.7%), overall performance decreased.

As shown in Table 2, the mandatory Meningococcal (77.3%) and Pneumococcal (79.7%) vaccines show lower performance. In contrast, performance accuracy was higher for vaccines such as Polio (84.3%), MMR (82.5%), Diphtheria (84.2%), and Hepatitis B (84.8%). Among the recommended vaccines, LLMs generally perform well for more common vaccines, such as Hepatitis A (88.0%), HPV (85.9%), and Influenza (89.3%). However, for less common vaccines like Dengue (72.7%), RSV (67.4%), and Shingles (78.7%), overall performance decreased.

**Table 2:** Comparison of overall accuracy (%) across large language models based on question vaccine type

Model		HPV	Covid-19	Flu	Hepatitis B	MMR	Shingles	Polio	Pneumococcal	Meningococca	Dengue	Diphtheria	RSV	Hepatitis A	Malaria
Flagship															
GPT-4.5		93.2%	87.2%	92.7%	88.2%	84.9%	84.0%	91.6%	83.2%	81.2%	79.9%	82.3%	87.9%	88.7%	86.4%
Llama-4-maverick		91.9%	88.7%	95.3%	88.8%	89.9%	90.5%	90.6%	84.9%	89.3%	84.2%	91.6%	88.3%	94.8%	89.5%
Deepseek-v3		93.0%	89.7%	93.2%	90.2%	89.4%	86.0%	88.2%	82.9%	87.7%	81.0%	93.6%	87.9%	94.8%	92.6%
Grok-3		90.1%	84.2%	89.8%	87.4%	88.0%	86.2%	86.4%	75.4%	83.8%	80.3%	84.3%	87.0%	91.1%	84.0%
Qwen2.5		92.6%	86.8%	92.4%	86.6%	87.8%	81.0%	86.6%	72.8%	74.8%	79.2%	81.5%	85.3%	85.4%	79.0%
GLM-4		85.6%	86.5%	89.4%	82.5%	80.7%	74.5%	84.0%	72.2%	76.4%	70.6%	81.5%	75.3%	90.1%	79.6%
Earlier															
GPT-3.5-Turbo		78.6%	83.6%	83.2%	77.8%	73.7%	72.1%	73.0%	72.5%	70.2%	66.7%	73.5%	67.5%	81.7%	82.7%
GPT-4		89.9%	86.7%	92.0%	90.2%	89.6%	79.5%	86.4%	76.2%	83.2%	80.3%	86.7%	61.5%	92.5%	84.6%
GPT-4o		91.9%	91.3%	92.4%	90.0%	91.7%	87.6%	91.3%	88.1%	89.6%	83.9%	90.0%	87.4%	96.2%	85.8%
Gemini-1.5-Pro		86.7%	71.9%	84.4%	78.3%	87.0%	74.0%	78.7%	78.6%	86.7%	74.9%	75.9%	84.4%	92.0%	82.1%
Claude-3-Opus		90.7%	84.9%	91.8%	86.4%	83.6%	79.8%	90.0%	77.1%	77.3%	71.7%	82.7%	81.0%	85.9%	84.6%
Yi-Lightning		80.6%	80.0%	87.2%	77.8%	79.5%	76.4%	77.7%	71.3%	78.3%	66.7%	79.9%	75.8%	87.3%	83.3%
Reka-Core		84.3%	87.4%	92.2%	84.8%	81.0%	79.5%	87.4%	75.7%	83.5%	73.5%	83.5%	78.4%	83.1%	89.5%
Overall		88.4%	85.3%	90.5%	85.3%	85.1%	80.9%	85.5%	77.7%	81.7%	76.4%	83.6%	80.6%	89.5%	84.9%

Inter-rater reliability was quantified using Cohen’s *κ*. Agreement was substantial across languages (English *κ* = 0.686; Spanish *κ* = 0.702; Chinese *κ* = 0.751; see Table 3), supporting the operational clarity and cross-linguistic consistency of the category framework. The remaining items were subsequently single-coded using the finalized codebook.

**Table 3:** Inter-rater reliability (Cohen’s *κ*) for category annotation across languages

Language	*N*	Raw agreement	Cohen’s *K*	Interpretation
English	80	73.8%	0.686	Substantial
Spanish	80	77.5%	0.702	Substantial
Chinese	82	81.7%	0.751	Substantial

In terms of question category, Misconceptions and Corrections had the highest proportion of correct answers (93.0%), while Types and Basic Information (82.5%), Cost and Accessibility (82.6%), and Effectiveness and Benefits (82.5%) had over 10% lower accuracy in comparison (Table 4).

**Table 4:** Comparison of overall accuracy (%) across large language models based on question category

Model	Dosing^a^	Effectiveness^a^	Safety^a^	Types^a^	Myths^a^	Prevention^a^	Cost^a^	Regulatory^a^	Other^a^
Flagship									
GPT-4.5	85.70%	90.70%	86.00%	85.70%	94.00%	90.20%	88.10%	87.00%	91.90%
Llama-4-maverick	89.30%	91.00%	90.70%	86.50%	96.20%	92.70%	87.60%	89.50%	97.00%
Deepseek-v3	86.80%	92.70%	89.80%	88.60%	95.70%	93.10%	92.00%	91.40%	92.90%
Grok-3	84.40%	88.10%	84.70%	83.60%	93.60%	90.70%	88.10%	88.30%	87.90%
Qwen2.5	83.10%	88.80%	83.60%	80.80%	96.70%	91.10%	85.60%	87.00%	85.90%
GLM-4	77.90%	83.90%	83.20%	80.50%	91.40%	91.50%	77.10%	87.70%	
Earlier									
GPT-3.5-Turbo	72.40%	76.80%	78.00%	76.80%	88.30%	85.00%	71.60%	84.60%	82.80%
GPT-4	84.50%	87.30%	85.60%	81.60%	95.20%	92.30%	75.60%	86.40%	89.90%
GPT-4o	89.60%	91.30%	91.10%	88.60%	94.80%	93.10%	82.60%	90.10%	94.90%
Gemini-1.5-Pro	79.30%	78.20%	79.70%	80.70%	90.50%	89.00%	81.10%	81.50%	88.90%
Claude-3-Opus	82.90%	86.90%	83.90%	81.00%	93.80%	86.60%	83.60%	87.70%	90.90%
Yi-Lightning	76.50%	79.30%	80.50%	76.50%	86.00%	85.00%	80.10%	85.20%	88.90%
Reka-Core	79.70%	86.70%	88.30%	81.50%	92.90%	89.40%	80.60%	87.00%	
Overall	82.50%	86.30%	85.00%	82.50%	93.00%	90.00%	82.60%	87.20%	90.20%

^a^Dosing = dosing and recommendations, Effective = effectiveness and benefits, Safety = safety and side effects, Types = types and basic information, Myths = misconceptions and corrections, Prevention = awareness, behavior, and prevention, Cost = cost and accessibility, Regulatory = regulatory and monitoring systems; Other = other.

Based on the generalized linear mixed model (GLMM) analysis, flagship models had a 1.57 higher odds (95% CI [1.50, 1.65], *P* < 0.001) of answer correctness compared to early models. Language influenced performance; Spanish (OR = 0.56, 95% CI [0.43, 0.72], *P* < 0.001) and Chinese questions (OR = 0.35, 95% CI [0.28, 0.45], *P* < 0.001) had a lower odds of correct answers compared to English. Few-shot prompting achieved 1.17 OR (95% CI [1.10, 1.24], *P* < 0.001) compared to zero-shot prompts, while CoT had 21% lower odds of correct answers (OR = 0.79, 95% CI [0.75, 0.84], *P* < 0.001). Correct answers to COVID-19, diphtheria, flu, hepatitis A, Malaria, and Polio did not differ significantly from HPV at *P* = 0.05. However, relative to HPV, Dengue, Meningococcal, MMR, Pneumococcal, and Shingles related questions had a significantly lower odds of correct answers (*P* < 0.001). Questions about awareness, behavior, and prevention (OR = 1.97, 95% CI [1.24, 3.13], *P* = 0.004), misconceptions and corrections (OR = 2.23, 95% CI [1.54, 3.23], *P* < 0.001), and regulatory and monitoring systems (OR = 1.78, 95% CI [1.02, 3.13], *P* = 0.044) were more likely to be answered correctly relative to the reference category (dosing and recommendations), while other categories showed no significant differences. The full model output is reported in Supplementary Table 2.

### Cross-language sensitivity and parallel subset analyses

**Fig. 4:**
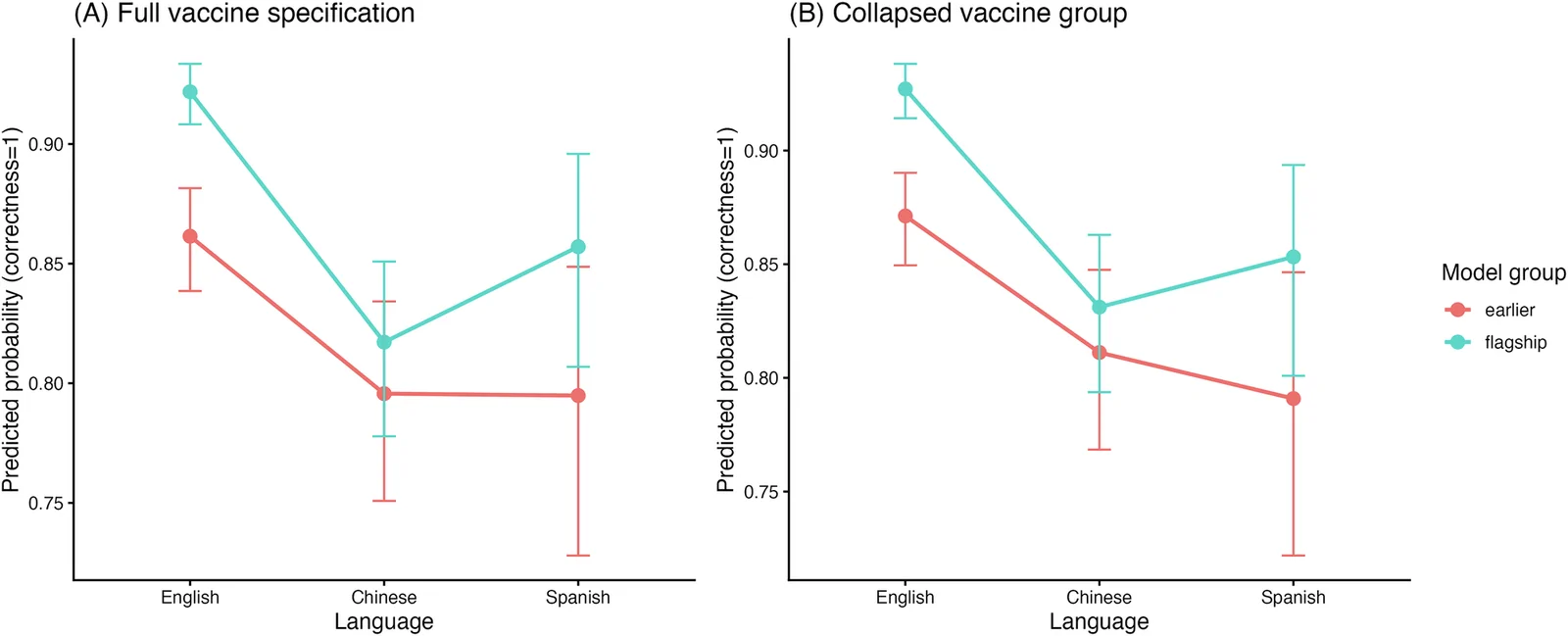
Sensitivity analysis restricting English to authority-derived items (Source One): predicted correctness by language and model group. **A** Full vaccine specification. **B** Collapsed vaccine group.

Within English items, questionnaire-derived questions were associated with lower correctness than authority-derived items (OR = 0.479, 95% CI 0.449–0.510; Supplementary Table 3), suggesting that heterogeneous item sources may attenuate cross-language contrasts in the full dataset. In the full dataset, cross-language differences were not statistically significant (Spanish vs English: OR = 0.772, *p* = 0.181; Chinese vs English: OR = 0.799, *p* = 0.124; Supplementary Table 4). After restricting the English subset to authority-derived items only, language effects became statistically significant: Spanish vs English OR = 0.634 (95% CI: 0.427–0.943, *p* = 0.024) and Chinese vs English OR = 0.616 (95% CI: 0.456–0.832, *p* = 0.002) (Table 5). Predicted probabilities were similar under the full and collapsed vaccine specifications, indicating robustness to alternative vaccine grouping (Fig. 4).

**Table 5:** Mixed-effects logistic regression (restricted English subset to authority-derived items)

Term	Restricted Model^a^ OR ^b^ (95% CI)	*P* value
Spanish vs English	0.634 (0.427–0.943)	0.024
Chinese vs English	0.616 (0.456–0.832)	0.002
Flagship vs Earlier	1.902 (1.773–2.041)	<0.001

^a^Restricted English subset includes Source One items only.

^b^ORs reported with 95% CIs.

On the parallel-language subset (*n* = 100), both GPT-4o and DeepSeek-V3 demonstrated high and broadly comparable accuracy across languages (Supplementary Table 5). Bootstrap 95% confidence intervals substantially overlapped for all language pairs, suggesting limited cross-lingual variability within aligned items. Paired McNemar tests comparing English and Chinese responses did not identify statistically significant differences for either model (all *p* > 0.05), indicating that cross-lingual discrepancies were small in magnitude. Discordant cases were infrequent (≤7% of items) and concentrated primarily in clinically nuanced domains such as safety and dosing, rather than distributed broadly across categories. For DeepSeek-V3, discrepancies were disproportionately concentrated in safety and dosing-related categories, whereas GPT-4o exhibited fewer discordant items overall. These findings suggest that language-related performance differences, when present, may be more sensitive in clinically nuanced domains rather than reflecting broad linguistic bias.

### Failure pattern analysis

From Table 6, nearly half of the incorrect responses (49%) were categorized as Overgeneralization, in which models provided vague or broadly applicable statements without applying vaccine-specific constraints. A substantial proportion (approximately one-third) reflected failures to correctly apply vaccination rules, particularly regarding dosing intervals, contraindications, and age eligibility. Please refer to Supplementary Table 6 for more details.

**Table 6:** Overall distribution of error categories among 150 sampled incorrect responses

Error category	Count	Percent (%)
Overgeneralization	74	49.3
Dosing interval	19	12.7
Contraindication	15	10
Other	14	9.3
Age group	14	9.3
Vaccine type	8	5.3
Regulatory program	2	1.3

Rule-specific failures were most commonly observed in questions requiring precise interval scheduling (e.g., multi-dose series), contraindication assessment (e.g., pregnancy, immunocompromised status), and age-based eligibility. The distribution of error types was broadly consistent across model backbones, with overgeneralization being the dominant failure mode in all three models. Example 1 (Dose interval): The model recommended a two-dose schedule instead of the required three-dose schedule for individuals aged ≥15 years. Example 2 (Contraindication): The model failed to identify pregnancy as a contraindication for a live attenuated vaccine.

## Discussion

This study establishes a large-scale multilingual benchmark (VaxEval) to systematically evaluate vaccine-related knowledge in contemporary LLMs. Across 1,886 multiple-choice questions spanning 14 vaccines and three UN languages—English, Spanish, and Chinese—we observed substantial heterogeneity in model performance. Flagship model generations significantly outperformed earlier versions, with nearly twofold higher odds of correctness in mixed-effects analyses. While overall accuracy exceeded 80% across languages, performance varied meaningfully by vaccine type, language, and question category. Common and widely discussed vaccines showed high accuracy (e.g., influenza 90.5%, hepatitis A 89.5%, HPV 88.4%), whereas performance dropped for several clinically nuanced or less commonly addressed vaccines, including RSV (80.6%), dengue (76.4%), and shingles (~80.9%), as well as for some routine childhood vaccines such as meningococcal (81.7%) and pneumococcal (77.7%) items. English items consistently achieved the highest accuracy, consistent with prior cross-language evaluations reporting variability in vaccination responses across linguistic contexts^15^, whereas Spanish and Chinese items were associated with lower odds of correct responses. Importantly, these cross-language differences persisted even after restricting the English dataset to authority-derived items, suggesting that dataset composition alone does not fully account for observed disparities. At the question-category level, misconception-correction and prevention-oriented items were more likely to be answered correctly than dosing and scheduling questions. This pattern aligns with prior findings showing that LLMs often perform well on myth-rebuttal style vaccine questions but exhibit inconsistency in structured clinical guidance^16^.

Parallel-language robustness analyses further clarified that when content was semantically aligned across languages, performance differences were attenuated and generally not statistically significant. While prior evaluations of ChatGPT on vaccine-related MCQs reported variable performance across infectious disease domains, these studies were largely limited to single-model or single-language settings^17^. Our findings indicate that apparent cross-language gaps likely reflect interactions between multilingual model capacity and dataset heterogeneity, rather than intrinsic linguistic bias.

Several patterns observed in this study provide insight into how LLMs retrieve and apply vaccine-related knowledge. *First, across tasks, performance appears to rely on mapping questions to relevant guideline rules, resembling a retrieval-and-selection process, with limited evidence of sustained multi-step reasoning*. Each MCQ requires identification of a specific rule—such as age eligibility, dose intervals, or contraindications—and selection of the corresponding answer from a fixed set. This task structure emphasizes accurate rule identification and rule–answer mapping, with limited requirement for integration across multiple inferential steps. Errors, therefore, appear to arise primarily from misapplication of these constrained rules, including confusion between closely related recommendations or incorrect handling of boundary conditions, more so than from gaps in general medical knowledge. *Second, performance varies with task demands*. Accuracy declines for dosing and scheduling questions requiring precise application of conditional rules (e.g., age, immunocompromised status, prior vaccination history) and is higher for misconception-correction and prevention-oriented items. Nearly half of the incorrect responses involved overgeneralization, with models defaulting to broadly applicable statements without applying vaccine-specific constraints; similar patterns have been observed in clinical examination benchmarks, where LLMs produce plausible but guideline-incomplete answers^5^. Rule-based failures—particularly involving dose intervals and contraindications—formed another major class of errors. It should be noted that the prominence of overgeneralization in our error taxonomy may partly reflect constraints of the MCQ format and prompt design, and not only intrinsic model behavior; constrained response options may systematically favor broadly applicable answers over guideline-specific ones. *Third, performance is sensitive to prompting strategy*. Few-shot prompting yielded modest improvements over zero-shot prompting, whereas CoT prompting reduced accuracy. This is consistent with evidence that CoT does not uniformly benefit performance and may degrade accuracy in constrained factual retrieval settings requiring precise application of structured clinical rules^18^. In this context, CoT introduces intermediate reasoning steps that increase the risk of overgeneralization or misapplication of vaccine-specific constraints; concise direct responses often outperform CoT on factual recall and classification tasks^19^. By contrast, few-shot prompting reinforces task structure and the mapping between rules and answer choices without imposing sequential reasoning, likely explaining its advantage. *Fourth, performance varies by representation in training data*. Accuracy is higher for widely discussed vaccines (e.g., COVID-19, HPV, influenza) and lower for clinically nuanced or less frequently represented vaccines. This pattern is consistent with differences in the distribution and prominence of vaccine-specific content in training corpora, which likely influence the reliability of retrieving guideline-concordant rules. Tasks involving less common vaccines or more specialized indications often require more precise distinctions (e.g., product-specific schedules, conditional recommendations), increasing susceptibility to confusion between similar rules or extrapolation from more familiar vaccines. Errors in these cases appear to reflect limitations in accessing sufficiently specific representations, rather than the absence of underlying medical knowledge. These patterns indicate that performance in this domain is governed less by reasoning depth than by the reliability of retrieving and correctly applying narrowly defined clinical rules. Future work should evaluate whether CoT confers advantages in open-ended vaccine question generation tasks, where multi-step reasoning may be more appropriate than in the constrained MCQ format evaluated here.

Multilingual evaluation of LLMs presents methodological challenges that extend beyond translation fidelity. In this study, English items were derived from both health-authority sources and peer-reviewed questionnaires, whereas Spanish and Chinese items were authority-derived and independently constructed rather than direct translations of English questions. Sensitivity analyses restricting English to authority-derived items reduced—but did not eliminate—cross-language differences, indicating that dataset composition partially moderates observed language effects. Parallel-language analyses further showed that when semantically aligned items were tested across languages, performance gaps narrowed and were not statistically significant. This suggests that a substantial portion of cross-language variability stems from differences in item difficulty, topical distribution, and source composition rather than intrinsic linguistic limitations alone, consistent with prior multilingual evaluation frameworks^20^. These findings highlight an important methodological implication: multilingual benchmarking must distinguish between language effects and dataset heterogeneity. Evaluations based on independently constructed datasets risk conflating linguistic performance with structural differences in item composition. English dominance in training data remains a plausible contributor to performance asymmetry^21,22^, underscoring the need for more diverse multilingual corpora and evaluation frameworks. Adaptive communication strategies tailored to cultural contexts may help mitigate these differences in applied settings^23^. In this study, “misinformation” is operationalized through misconception-correction items (e.g., common myths and evidence-based rebuttals) rather than real-world detection of misleading narratives in open text. Future work should evaluate open-ended responses, citation behavior, and failure modes such as confident but incorrect explanations.

From an application perspective, the high—but imperfect—accuracy observed across models suggests potential for supporting vaccine information delivery, consistent with feasibility studies indicating that AI chatbots may enhance public health education, although robust evaluation remains limited^24^; however, it also highlights risks. Even modest error rates are consequential in vaccine contexts, particularly for dosing schedules, contraindications, and age eligibility. In public-facing settings, users may infer reliability from fluent responses and act on incorrect guidance. Errors may plausibly affect behavior through missed vaccination opportunities (e.g., inappropriate deferral due to incorrect interval advice), inappropriate vaccination (e.g., failure to identify contraindications), or reinforcement of hesitancy when incorrect risk statements are presented authoritatively. These risks are concentrated in schedule- and eligibility-sensitive domains and may be amplified by user overreliance on confident outputs. A recent scoping review highlights both the promise and methodological heterogeneity of chatbot-based vaccine literacy interventions^25^.

Translating these findings into actionable guidance, we identify model-level and deployment-level guardrails. The concentration of errors in dosing, contraindication, and age-eligibility domains—together with observed cross-language differences—points to three priorities for mitigation. First, as nearly half of incorrect responses reflected overgeneralization, retrieval-augmented generation grounded in jurisdiction-specific immunization guidelines may reduce this failure mode and improve schedule fidelity. Second, given the concentration of rule-based failures in dose intervals and contraindications, structured knowledge representations—such as machine-readable vaccine schedule tables or rule-based constraints—could be integrated to enforce high-risk factual requirements. Third, because residual errors persist in clinically sensitive domains despite high overall accuracy, guardrail mechanisms—including uncertainty signaling and human-in-the-loop escalation for individualized or clinical queries—remain essential to mitigate downstream harm. From a governance perspective, benchmarking should extend beyond aggregate accuracy to include domain-specific error characterization and multilingual robustness testing. As LLMs are increasingly used for health-related queries, equitable access to high-performing systems and transparent evaluation standards will be essential. Continuous benchmarking across vaccine types, languages, and demographic contexts will be critical to ensure that advances in AI capability translate into reliable and globally applicable health communication tools.

This study has several strengths. First, we introduce a large-scale, multilingual vaccine knowledge benchmark comprising 1,886 MCQs across 14 vaccine types and three UN languages. Compared with prior evaluations relying on small question sets, this design enables more stable estimation of model performance across vaccine domains, prompting strategies, and languages. Second, we integrate hierarchical mixed-effects modeling, sensitivity analyses, and parallel-language robustness testing to disentangle model generation effects from dataset composition and linguistic variation. Third, structured error taxonomy analysis provides insight into systematic failure modes beyond aggregate accuracy metrics. Nevertheless, several limitations warrant consideration: although the benchmark represents the largest curated vaccine MCQ dataset to date, question distribution across vaccine types is uneven. Vaccines such as RSV, dengue, and malaria were represented by relatively few items, whereas HPV and COVID-19 were more extensively covered. Although this distribution reflects the relative breadth of available authoritative guidance and peer-reviewed evidence at the time of curation, it may influence performance estimates and limit generalizability to underrepresented vaccines. In addition, multilingual datasets were independently constructed rather than strictly parallel (i.e., not exact translations of the same questions), which may introduce systematic differences in item difficulty independent of linguistic factors. To address this, we conducted prespecified sensitivity analyses and parallel-language robustness checks using semantically aligned content. While these approaches enhance comparability, residual confounding cannot be fully excluded. Additionally, MCQ-based evaluation assesses factual accuracy within constrained response formats and does not capture open-ended reasoning quality, source attribution, or real-world conversational dynamics. Accordingly, benchmark accuracy should not be construed as evidence of clinical reliability or readiness for real-world deployment—such as patient counseling, clinical decision support, or public health communication—without rigorous prospective validation in operational settings and comprehensive, context-specific safety evaluation. Cross-language comparisons of vaccine knowledge among LLMs reflect both multilingual model capability and underlying dataset composition, as the Spanish and Chinese items were independently constructed rather than direct translations of English items. To better isolate linguistic effects, we therefore analyzed a smaller parallel-language subset while retaining the full benchmark to preserve real-world heterogeneity and reflect the practical challenges of multilingual LLM performance. A fully parallelized benchmark would reduce content heterogeneity but would require substantial translation resources and may introduce translation drift. In addition, the use of standardized prompts across all models and languages enables fair and reproducible comparison but may not reflect each model’s optimal prompting conditions; performance estimates, particularly for earlier-generation models, should therefore be interpreted as lower bounds under a fixed evaluation protocol rather than each model’s peak capability. Finally, while temperature was set to 0 to promote reproducibility, deterministic outputs cannot be fully guaranteed due to other stochastic components in model inference pipelines, such as sampling in top-k or top-p decoding layers. We recognize the rapid evolution of LLM architectures; the specific models used in the VaxEval assessment may be superseded by the time of publication. This study establishes a transparent and reproducible framework for benchmarking vaccine knowledge models against prespecified performance criteria. The models met these criteria and demonstrated stable performance across evaluation metrics, supporting the technical feasibility of LLM-assisted vaccine safety monitoring and timely dissemination of vaccine-related information. These findings provide an empirical foundation for strengthening epidemiological signal evaluation, large-scale social listening, and public health communication. However, integration into clinical workflows must remain contingent upon structured governance, including rigorous auditability, longitudinal performance monitoring, and unfiltered human-led oversight. We therefore report evaluation dates and model identifiers (Table 7) and release prompt templates and scoring scripts to support independent re-evaluation on the same benchmark under current model versions.

Future research, situated within a global health framework, should move beyond multiple-choice benchmarks to evaluate open-ended generation, explanation calibration, and citation reliability in vaccine-related responses. A practical pathway from benchmark accuracy to deployable vaccine chatbots involves several intermediate steps. First, developers should define the intended user group (lay public vs clinicians) and triage risk level, because dosing/contraindication errors carry higher potential harm than general educational content. Second, VaxEval can inform base-model selection (including multilingual robustness) and identify high-risk domains (e.g., schedules, eligibility, contraindications) that require additional constraints. Third, chatbot responses should be grounded using retrieval-augmented generation (RAG) over jurisdiction-specific immunization guidelines with document-level provenance and periodic guideline refresh. Fourth, high-risk logic should be enforced with structured representations (e.g., machine-readable schedule tables or rule-based validators) to prevent overgeneralization and interval/eligibility errors observed in our taxonomy. Fifth, deployment should include safety layers—uncertainty signaling, refusal or escalation for individualized clinical scenarios, and human-in-the-loop review for edge cases. Sixth, future work should also compare medically tailored LLMs and clinically grounded retrieval systems with general-purpose models under identical evaluation protocols, particularly for guideline-sensitive tasks (e.g., contraindications and dosing schedules). Establishing human baselines—by having vaccination experts and lay participants complete matched VaxEval subsets—would contextualize model performance, quantify item difficulty, and identify domains where LLM outputs most diverge from human judgment. Finally, systems should be evaluated iteratively using held-out benchmark items plus targeted stress tests for dosing and contraindications, followed by prospective user studies assessing comprehension, trust calibration, and behavioral impact. For epidemic response settings, it is particularly critical to assess whether models appropriately communicate uncertainty, align with evolving authoritative guidance, and avoid overconfident or outdated recommendations. Expanding inclusion of low-resource languages and region-specific immunization policies would strengthen global applicability, especially in outbreak-prone and resource-constrained settings where multilingual communication gaps can undermine response efforts. Methodologically, next-generation benchmarks should incorporate larger parallel-language corpora and more balanced representation across vaccine types to better disentangle linguistic performance from dataset heterogeneity. Prospective evaluation studies—including pragmatic or randomized trials of vaccine chatbots—are needed to assess behavioral, clinical, and public health impact^26^.

In this large-scale multilingual evaluation of contemporary LLMs, newer flagship systems demonstrated measurable improvements in vaccine-related knowledge accuracy, yet domain-specific and cross-language variability persists. Performance disparities across vaccine types and question categories reveal structured blind spots, particularly in dosing schedules and rule-based constraints. Cross-language differences appear to reflect both multilingual modeling capacity and dataset composition effects, underscoring the importance of careful benchmark design when interpreting global AI performance. While overall accuracy exceeded 80%, residual error rates—especially in clinically nuanced domains—necessitate cautious integration of generative systems into vaccine information pathways. Continued benchmarking, multilingual dataset expansion, and structured governance mechanisms will be essential to ensure that advances in LLMs translate into equitable, reliable, and contextually appropriate vaccine communication support.

## Methods

### Study design and model evaluation framework

We evaluated 13 LLMs spanning major providers: GPT-4o, GPT-4.5, GPT-4, GPT-3.5-Turbo (OpenAI/ChatGPT), Claude 3 Opus (Anthropic), Gemini 1.5-Pro (Google), Llama-4 Maverick (Meta), Grok-3 Fast (xAI), Qwen-2.5-72B-Instruct (Alibaba), GLM-4 (Zhipu), DeepSeek-V3 (DeepSeek), Reka Core (Reka), and Yi-Lightning (01.AI)^8,9,11,13,27–35^. Models were grouped by evaluation wave: earlier (October 2024) and flagship (July 2025)^36^.

In subsequent assessments, models were compared based on the two dates of evaluation. Newer model releases evaluated in July 2025 were considered “flagship” (*n* = 6), while older models evaluated in October 2024 were “earlier” (*n* = 7) versions, including older releases from the same company (e.g., OpenAI).

**Fig. 5:**
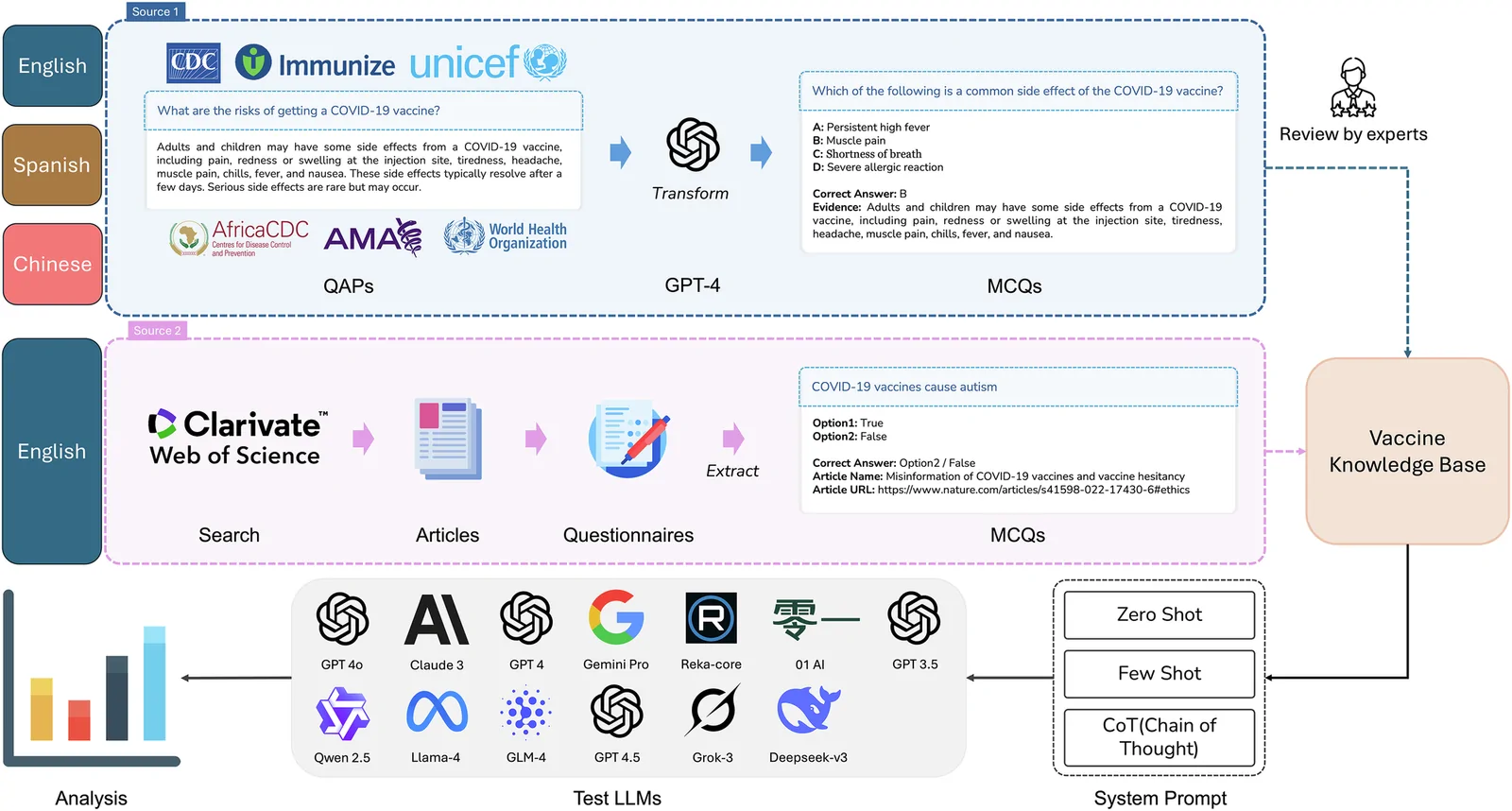
Vaccine knowledge base: construction and LLM processing pipeline.

Figure 5 illustrates the study design, detailing how the datasets were independently collected, processed, and cleaned from the two main sources. Utilizing this dataset, the study evaluates the performance of the selected LLMs in answering vaccination MCQs accurately. The prompt template used for generating MCQs is provided in Supplementary Fig. 1.

We evaluated three commonly used prompting settings—zero-shot, few-shot, and CoT—to reflect realistic deployment patterns and to test whether additional guidance improves factual option selection under a fixed multiple-choice format^37–39^. Zero-shot represents minimal user prompting; few-shot tests whether providing in-context exemplars improves adherence to task format and factual selection; and CoT tests whether instructing step-by-step reasoning improves performance, while noting that such reasoning instructions may not reflect typical public use and are evaluated here as an experimental condition.

To ensure fair and consistent comparisons across LLMs, we used a fixed system instruction and standardized user templates across all models within each language, and implemented a custom post-processing script to extract a single option letter (A–D) from each response regardless of format. Empty or null outputs occurred in 1.64% of all model–item queries and were counted as incorrect. An example of the few-shot/zero-shot/Cot prompting approach is illustrated in Supplementary Fig. 1. Model responses varied in formatting, and some failed to follow the requested JSON structure.

We further applied structured validation and quality-control procedures. For the benchmark items, we removed duplicates, screened options for plausibility, and verified answer keys against source texts; ambiguous items were revised or excluded by a public-health reviewer. For model outputs, a public-health professional audited extracted answers, and any invalid extractions were manually checked and adjudicated as needed.

All API-based models were queried via official provider endpoints, while open-weight models were run on cloud instances with NVIDIA V100 GPUs (Table 7). No model was fine-tuned, retrained, or updated using the curated vaccine dataset; all evaluations were conducted on publicly available model versions under standardized inference conditions. Each model–question instance was evaluated in an isolated session (no conversation history) with deterministic decoding when available (e.g., temperature = 0), and model/version identifiers were recorded. Exact-match accuracy was defined as the proportion of questions for which the extracted option matched the verified reference answer^40^. Models were evaluated within two predefined windows (October 2024 and July 2025; Table 7). Because API-served models may be updated or deprecated, results are reported as performance snapshots at the time of evaluation. Prompt templates and scoring scripts are released to enable independent re-evaluation.

**Table 7:** Description of model families used in the performance evaluation, including official public release dates and the date of evaluation conducted in this study

Service/Family	Model	Statusa^a^	Release date	Evaluation date	Cohort
ChatGPT	GPT-3.5-Turbo	Deprecated	Nov-22	Oct-24	Earlier
ChatGPT	GPT-4	Deprecated	Mar-23	Oct-24	Earlier
ChatGPT	GPT-4o	Active	May-24	Oct-24	Earlier
ChatGPT	GPT-4.5	Active	February 2025	Jul-25	Flagship
Gemini	Gemini-1.5-Pro	Deprecated	May-24	Oct-24	Earlier
Llama	Llama-4-maverick	Deprecated	Apr-25	Jul-25	Flagship
Claude	Claude-3-opus	Active	Mar-24	Oct-24	Earlier
Deepseek	Deepseek-v3	Deprecated	Dec-24	Jul-25	Flagship
xAI	Grok-3	Deprecated	February 2025	Jul-25	Flagship
Qwen	Qwen-2.5-72b-instruct	Deprecated	Jul-25	Jul-25	Flagship
GLM	GLM-4	Active	Jun-24	Jul-25	Flagship
Reka AI	Reka-core	Deprecated	Apr-24	Oct-24	Earlier
01.AI	Yi-lightning	Active	Oct-24	Oct-24	Earlier

^a^Status reflects provider availability on Oct 15, 2025.

Annotation and source verification were conducted by at least two Master’s-level researchers (MPH/MSc or equivalent) per language, all of whom completed advanced training in vaccinology and vaccine confidence, including Croucher Summer Courses in Vaccinology and/or Vaccine Hesitancy. Annotation frameworks, adjudication, and quality assurance were overseen by doctoral-level investigators with expertise in vaccine confidence research.

### Dataset construction

**Fig. 6:**
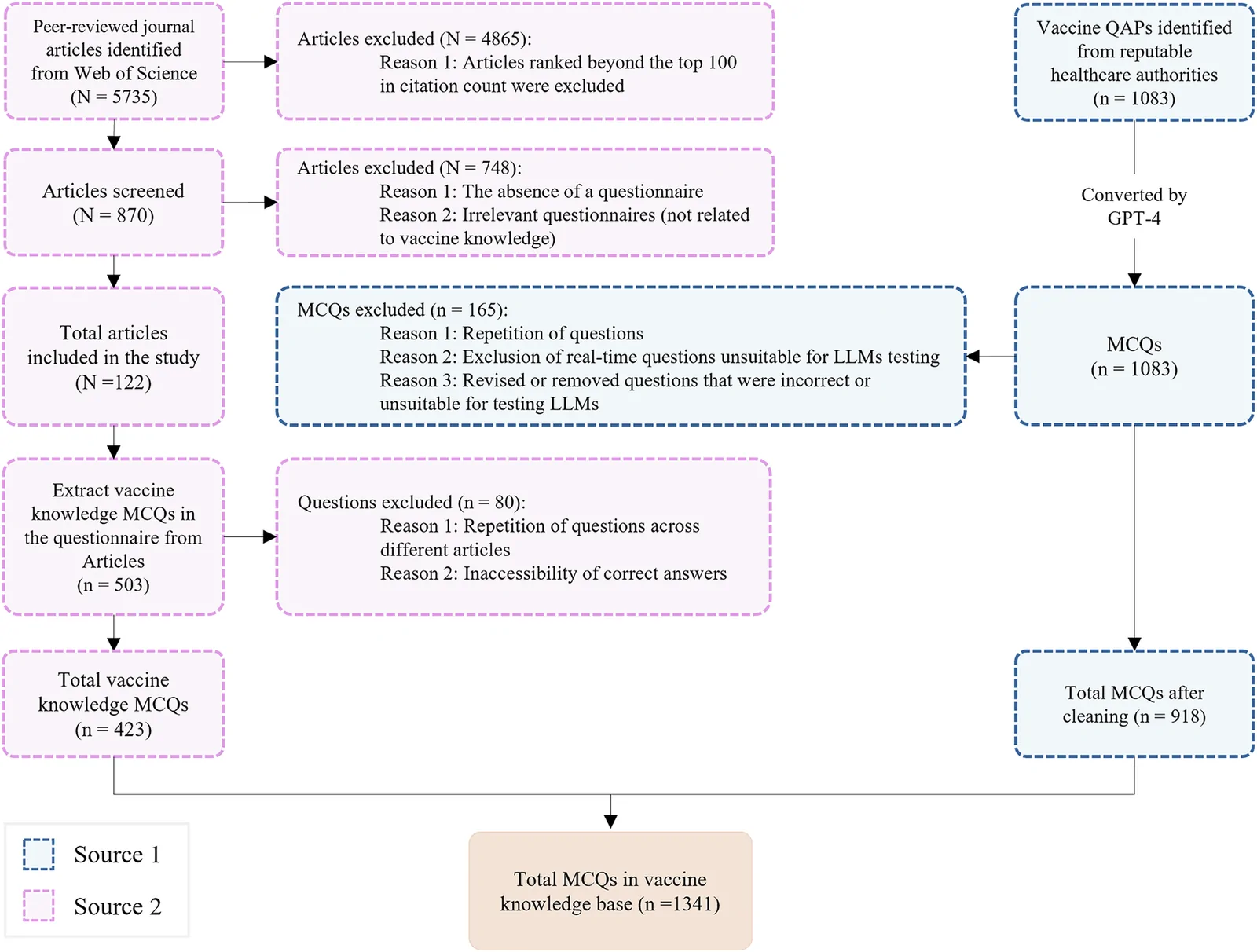
Flow chart of English vaccine knowledge base construction.

Our dataset comprises MCQs in three UN languages: English, Spanish, and Chinese. Each language-specific dataset includes MCQs focused on 14 common vaccines: *COVID-19, Dengue, Diphtheria, Hepatitis A, Hepatitis B, Human Papillomavirus (HPV), Influenza, Malaria, Measles, Mumps, and Rubella (MMR), Pneumococcal, Meningococcal, Polio, Respiratory Syncytial Virus (RSV), and Shingles*. Selection was guided by expected public-health relevance, frequency of guidance updates, and anticipated public query volume in multilingual settings. The dataset compiles information from two main sources: knowledge bases from leading global health authorities and peer-reviewed literature. Figure 6 provides a detailed explanation of the integration process for the English knowledge database, with a similar process as Source 1 applied to Spanish and Chinese datasets.

We used multiple-choice questions to enable scalable, reproducible evaluation with unambiguous scoring across models and languages. MCQs reduce subjectivity relative to free-text grading and support item-level statistical modeling. However, MCQs do not capture risks unique to open-ended generation (e.g., hallucinated details, misleading framing, or omission of critical safety caveats); we therefore treat MCQ accuracy as an upper-bound proxy for factual retrieval rather than a complete measure of clinical safety.

Source One (knowledge base from global health authorities) incorporates data from leading global health and healthcare authorities, including the American Medical Association (AMA), Africa Centers for Disease Control and Prevention (Africa CDC), Centers for Disease Control and Prevention (CDC), Immunize.org, United Nations International Children’s Emergency Fund (UNICEF), and World Health Organization (WHO), reflecting global perspectives from countries where these entities operate.

For the English dataset, we first curated 1083 question-answer pairs collected from authoritative healthcare websites, then employed GPT-4 to transform them into MCQs, as GPT-4 demonstrated its capability to generate high-quality MCQs with one correct option and three distractors^41,42^. Following automated generation, we removed 133 duplicates and had a public health expert further refine the set by revising or discarding 32 inaccurate or inappropriate questions. This resulted in a high-quality English dataset comprising 918 MCQs. We subsequently applied the same methodology to curate Spanish and Chinese datasets, using native speakers in both languages to review the final datasets. The Spanish dataset, compiled from comparable health authority sources, consisted of 250 MCQs. Similarly, the Chinese dataset, sourced from global healthcare authorities such as the WHO and CDC, as well as national and regional government institutions, yielded 296 MCQs.

Source Two (vaccination knowledge questions from peer-reviewed journal articles) performed a comprehensive search of the Web of Science database for articles published from the past decade, from January 1, 2015, to April 2024, employing systematically selected keywords (Supplementary Fig. 2) derived from an extensive review of relevant literature. For each vaccine, the search string combined (i) vaccine names, common synonyms, and abbreviations (e.g., “COVID-19 vaccine,” “HPV,” “MMR,” “influenza vaccination”), with (ii) terms related to knowledge assessment and survey instruments (e.g., “knowledge,” “misinformation,” “misconception,” “survey,” “questionnaire,” “attitudes”), using Boolean operators to capture questionnaire-based studies assessing vaccine knowledge.

**Fig. 7:**
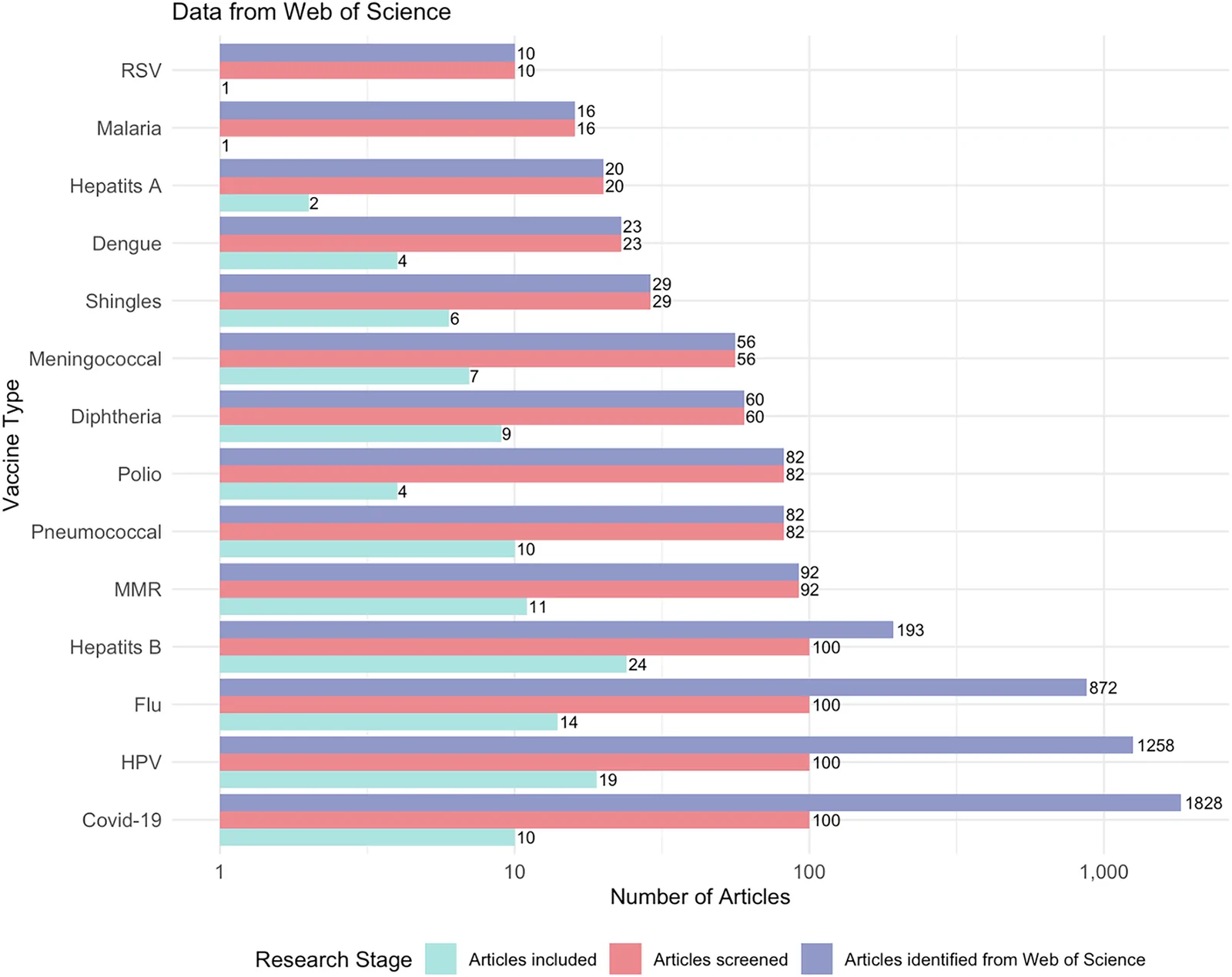
Number of articles by vaccine type and screening stage.

This initial search strategy yielded 5735 articles. We then applied a targeted sampling strategy: for vaccine types associated with more than 100 articles, we included the top 100 most-cited publications; for those with fewer than 100 articles, all retrieved studies were retained. This strategy yielded 870 eligible articles, which underwent manual screening. Figure 7 details the number of articles retained at each screening stage, organized by vaccine type.

Upon screening, we excluded 748 articles that lacked an appropriate questionnaire, such as articles with questionnaires unavailable or irrelevant to the study objective (e.g., questions solely focused on subjective vaccine-related attitudes). From the remaining 122 articles, 503 MCQs were collected from their questionnaires. The selected questions were standardized to a uniform format. Correct answers were determined using the papers’ supplementary materials or by consulting authorities like the CDC and WHO when necessary. Questions without provided answers or those not verifiable through public health authorities were excluded, and duplicates were removed. Ultimately, 422 unique multiple-choice questions were obtained. Source 2 includes only English-language datasets due to the limited availability of validated questionnaires in other languages.

### Question category coding and reliability assessment

We developed an a priori codebook defining eight thematic categories (dosing/recommendations; effectiveness/benefits; safety/side effects; types/basic information; misconceptions/corrections; awareness/behavior/prevention; cost/accessibility; regulatory/monitoring) plus “Other.” Each category included explicit inclusion and exclusion rules as well as illustrative examples (Supplementary Note 1).

For each language, two native-proficiency annotators independently assigned categories (Inter-rater) to a stratified random sample of questions (approximately 10–20% per language; *n* = 80 for English and Spanish, *n* = 82 for Chinese). All annotators held Master’s-level qualifications (MPH/MSc or equivalent) with advanced training in vaccinology; adjudication was overseen by doctoral-level investigators (see Study Design for full annotator details). Each item was assigned a single primary category. Discrepancies were resolved through adjudication by a senior public health researcher.

### Statistical analysis

To comprehensively evaluate overall performance across diverse languages, vaccine categories, and prompting strategies, a weighted-average approach was employed, with weights assigned according to the number of questions per respective category (e.g., Source, Language). Initial computations of accuracy and weighted averages were conducted using Microsoft Excel. For further analysis, a GLMM was used to assess the impact of models and question types on the correctness of answers. Setting correctness (binary 1,0) of each question as the dependent variable, model type (flagship, earlier), prompting method (zero-shot, few-shot, CoT), language (English, Spanish, and Chinese), and vaccine type in question (*n* = 14) were explored as fixed effects of the model. Question ID was used as a random effect. Odds ratios (ORs) were reported. In sensitivity analyses, we additionally adjusted for item source (authority vs peer-reviewed questionnaire) and restricted English to authority-derived items to improve comparability across languages. All analyses were conducted using R (v4.2.3) and Python (v3.11).

To evaluate the stability of cross-language findings and assess potential confounding due to dataset composition, we conducted predefined sensitivity analyses. (1) We adjusted the mixed-effects model for item source (Source 1 vs Source 2). (2) To improve cross-language comparability, we restricted the English dataset to authority-derived items only, matching the construction pipeline used for Spanish and Chinese datasets. Model estimates from the full dataset and restricted dataset were compared side by side. (3) We examined alternative vaccine grouping specifications (mandatory vs recommended) to evaluate whether vaccine-level findings were robust to classification structure. Results from these analyses are reported alongside the primary model to assess the consistency of language, model generation, and vaccine type effects.

To isolate linguistic effects from content heterogeneity, we constructed a parallel-language subset consisting of 100 semantically aligned questions translated into English, Spanish, and Chinese. Each question preserved identical content across languages. Paired comparisons were conducted using bootstrap confidence intervals and McNemar tests. This subset analysis was conducted only for zero-shot prompting to maintain comparability across models.

To characterize systematic failure modes beyond aggregate accuracy metrics, we conducted a structured error analysis focusing on high-performing models. From the three highest-performing models in the overall benchmark, we randomly sampled 50 incorrect responses per model (total *n* = 150). Errors were categorized using a predefined taxonomy comprising eight mutually exclusive categories: Dosing_Interval, Contraindication, Age_Group, Vaccine_Type, Regulatory_Program, Temporal_Policy, Overgeneralization, and Other (Supplementary Note 2).

Initial categorization was assisted by automated pre-labeling using an LLM-based classifier to improve efficiency. All labels were subsequently manually reviewed and adjudicated by two authors with public health expertise. Discrepancies were resolved through discussion until consensus was reached. We report the overall distribution of error categories, as well as stratified breakdowns by model backbone, language, and prompting strategy to explore whether certain failure patterns are systematically associated with specific evaluation conditions.

## Supplementary information

Supplementary information

## Data Availability

The finalized VaxEval multilingual benchmark dataset is publicly available via Zenodo (https://doi.org/10.5281/zenodo.18637915). The category codebook and prompt templates used in this study are provided in the Supplementary Information. Additional study materials may be made available to academic, non-commercial researchers upon reasonable request to the corresponding author, subject to a formal Data Use Agreement and any applicable licensing or institutional approvals.

## Code availability

The full codebase used for MCQ generation, model evaluation, scoring, and GLMM-based statistical analysis is publicly available at GitHub (https://github.com/perrychen1405-boop/VaxEval-code). A versioned release snapshot has been archived in Zenodo (v1.0.1; https://doi.org/10.5281/zenodo.18744748) to ensure long-term preservation and reproducibility. The repository is distributed under the MIT License.
