# Supplementary material for: Evaluating large language models on multilingual vaccine knowledge: a benchmark study
Source: NPJ Vaccines. 2026 Jun 10;11:187. doi: 10.1038/s41541-026-01500-1 (PMC13642938; doi:10.1038/s41541-026-01500-1)
Supplement: Supplementary file 1 [file 41541_2026_1500_MOESM1_ESM.pdf]

Supplementary

System Prompt for Generating MCQs by GPT4

System Prompt: "You are a learning engineer support bot specializing in developing high-quality multiple-choice questions (MCQs) for educational assessments. Your task is to create MCQs that effectively test a student's understanding of specific knowledge about vaccines. Each MCQ should be composed of the following three components: a stem, a Correct Answer, and three Distractors. There must always be only one correct answer and only three distractors.

Please structure the MCQ as follows: a clear, concise question stem, followed by four options labeled A, B, C, and D. Ensure the content of the question accurately reflects the details from the provided information. Please provide the response in the following JSON format:{ "question": "","options":[{"A": "","B": "","C": "","D": ""}],"correct_answer": "","evidence": ""}"

User Prompt: "Question: Do all COVID-19 vaccines protect against virus variants?
Answer: Yes, all WHO emergency-use listed or prequalified COVID-19 vaccines provide protection against severe disease and death from circulating COVID-19 variants. Any of the approved COVID-19 vaccines can be used either for the initial series or revaccination. "

Prompt template for choice questions

To assess the language model's understanding of vaccine-related topics, you will be provided with a multiple-choice question. After reading the question, please indicate the most correct answer by responding with a single letter (A, B, C, or D) that corresponds to the best option. Your response should be formatted as a JSON object with the key "options" followed by the chosen letter as the value. For instance, if you believe that option A is the correct answer, you should respond with:{{"options": "A"}}.Here is the multiple-choice question:{QAPs} Please select the best answer and respond in the JSON format provided above.

Few Shot prompt template

'''To assess the language model's understanding of vaccine-related topics, you will be provided with a multiple-choice question. After reading the question, please indicate the most correct answer by responding with a single letter (A, B, C, or D) that corresponds to the best option. Your response should be formatted as a JSON object with the key "options" followed by the chosen letter as the value. For instance, if you believe that option A is the correct answer, you should respond with:`{{"options": "A"}}`.Here is the multiple-choice question:{qa} Please select the best answer and respond in the JSON format provided above.'''
qa = {
Question: Do all COVID-19 vaccines protect against virus variants?
Option A: No, only specific vaccines provide protection against virus variants.
Option B: Yes, all COVID-19 vaccines provide protection against severe disease and death from circulating COVID-19 variants.
Option C: No, COVID-19 vaccines do not provide protection against virus variants.
Option D: Yes, but only if you receive multiple doses of the vaccine.
}

CoT prompt template

'''I need you to evaluate the following multiple-choice question about vaccines. Please analyze and select the correct answer using a systematic, evidence-based approach, maintaining consistent depth of analysis and accuracy regardless of the language in which the question is presented.

Step 1: Generate Initial Assessment
Carefully read the question and identify key vaccine-related concepts, terms, and the specific knowledge being assessed. Pay attention to any nuances or implied details in the question. Provide an initial comprehensive analysis of what the question is asking.

Step 2: Break Down Each Option Into Factual Claims
For each option, list the specific factual claims it contains in bullet point format. Identify claims related to:
- Medical mechanisms
- Statistical information
- Study results
- Dates and timelines
- Causal relationships
Respond only with the statements in the form of bullet points, nothing else.

Step 3: Generate Verification Queries
For each factual claim identified, list specific search queries that could be used to verify its alignment with scientific consensus and safety guidelines. These queries should target authoritative medical sources (e.g., CDC, WHO, peer-reviewed journals). Respond only with the queries in the form of bullet points, nothing else.

Step 4: Evaluate Evidence Quality
For each option, assess:
- Alignment with current scientific consensus
- Quality of supporting evidence
- Presence of any conflicting studies or evidence
- Contextual factors (regional guidelines, historical context if relevant)
When there is no clear consensus on a topic, clearly state that fact.

Step 5: Final Answer with Justification
Based on your analysis:
1. State the correct option clearly
2. Provide a detailed justification for why this option is correct
3. Explain why each incorrect option is inaccurate
4. Address any areas of uncertainty or conflicting evidence
5. Begin your answer with a description of any conditions or context relevant to the question
Your response should be formatted as a JSON object with the key "options" followed by the chosen letter as the value. For instance, if you believe that option A is the correct answer, you should respond with:`{{"options": "A"}}`.Here is the multiple-choice question:{qa} Please select the best answer and respond in the JSON format provided above.'''
qa = {
Question: Do all COVID-19 vaccines protect against virus variants?
Option A: No, only specific vaccines provide protection against virus variants.
Option B: Yes, all COVID-19 vaccines provide protection against severe disease and death from circulating COVID-19 variants.
Option C: No, COVID-19 vaccines do not provide protection against virus variants.
Option D: Yes, but only if you receive multiple doses of the vaccine.
}

Zero Shot prompt template

'''Here is the multiple-choice question:{qa}.Your response should be formatted as a JSON object with the key "options" followed by the chosen letter as the value. For instance, if you believe that option A is the correct answer, you should respond with:`{{"options": "A"}}`.'''
qa = {
Question: Do all COVID-19 vaccines protect against virus variants?
Option A: No, only specific vaccines provide protection against virus variants.
Option B: Yes, all COVID-19 vaccines provide protection against severe disease and death from circulating COVID-19 variants.
Option C: No, COVID-19 vaccines do not provide protection against virus variants.
Option D: Yes, but only if you receive multiple doses of the vaccine.
}

Supplementary Figure 1. System and user prompts for generating MCQs by GPT-4, with English template and example few-shot/zero-shot/CoT prompt.

Search Keywords

COVID-19: TS=(COVID or SARS-CoV-2) AND TS=(vaccin*) AND TS=(knowledge or misinformation or misconception* or rumo?r*) AND TS=(survey* or questionnaire*)

Dengue: TS=("Dengue Vaccines" OR "Dengue Vaccin*" OR "Dengvaxia") AND TS=(knowledge or misinformation or misconception* or rumo?r*) AND TS=(survey* or questionnaire*)

Diphtheria: TS=("Diphtheria Toxoid" OR "Diphtheria Vaccin*" OR "DTP Vaccin*" OR "DTaP Vaccin*" OR "Td Vaccin*" OR "Tdap Vaccin*") AND TS=(knowledge or misinformation or misconception* or rumo?r*) AND TS=(survey* or questionnaire*)

Hepatitis A: TS=("Hepatitis A Vaccines" OR "Hepatitis A Vaccin*" OR "Hep A Vaccin*" OR "HAV Vaccin*") AND TS=(knowledge or misinformation or misconception* or rumo?r*) AND TS=(survey* or questionnaire*)

Hepatitis B: TS=("Hepatitis B Vaccines" OR "Hepatitis B Vaccin*" OR "Hep B Vaccin*" OR "HBV Vaccin*") AND TS=(knowledge or misinformation or misconception* or rumo?r*) AND TS=(survey* or questionnaire*)

HPV: TS=("Papillomavirus Vaccines" OR "HPV Vaccin*" OR "Human Papillomavirus Vaccin*" OR "Bivalent HPV Vaccin*" OR "Quadrivalent HPV Vaccin*" OR "Nonavalent HPV Vaccin*" OR "Gardasil" OR "Cervarix") AND TS=(knowledge or misinformation or misconception* or rumo?r*) AND TS=(survey* or questionnaire*)

Influenza: TS=("Influenza Vaccines" OR "Influenza Vaccin*" OR "Flu Vaccin*" OR "Seasonal Influenza Vaccin*") AND TS=(knowledge or misinformation or misconception* or rumo?r*) AND TS=(survey* or questionnaire*)

Malaria: TS=("Malaria Vaccines" OR "RTS,S" OR "Malaria Vaccin*") AND TS=(knowledge or misinformation or misconception* or rumo?r*) AND TS=(survey* or questionnaire*)

MMR: TS=("Measles-Mumps-Rubella Vaccine" OR "MMR Vaccine" OR "MMR Vaccin*" OR "Measles-Mumps-Rubella Vaccin*" OR "Measles Vaccin*" OR "Mumps Vaccin*" OR "Rubella Vaccin*") AND TS=(knowledge or misinformation or misconception* or rumo?r*) AND TS=(survey* or questionnaire*)

Pneumococcal: TS=("Pneumococcal Vaccines" OR "Pneumococcal Vaccin*" OR "PPSV") AND TS=(knowledge or misinformation or misconception* or rumo?r*) AND TS=(survey* or questionnaire*)

Meningococcal: TS=("Meningococcal Vaccines" OR "Meningococcal Vaccin*" OR "MenACWY" OR "MenB" OR "Meningococcal B Vaccin*" OR "Meningococcal Polysaccharide Vaccin*") AND TS=(knowledge or misinformation or misconception* or rumo?r*) AND TS=(survey* or questionnaire*)

Polio: TS=("Poliovirus Vaccine, Inactivated" OR "Poliovirus Vaccine, Oral" OR Polio Vaccin* OR Poliomyelitis Vaccin*) AND TS=(knowledge or misinformation or misconception* or rumo?r*) AND TS=(survey* or questionnaire*)

RSV: TS=("Respiratory Syncytial Virus Vaccines" OR "RSV Vaccin*" OR "Respiratory Syncytial Virus Vaccin*") AND TS=(knowledge or misinformation or misconception* or rumo?r*) AND TS=(survey* or questionnaire*)

Shingles: TS=("Herpes Zoster Vaccine" OR "Shingles Vaccin*" OR "Zostavax" OR "shringrix") AND TS=(knowledge or misinformation or misconception* or rumo?r*) AND TS=(survey* or questionnaire*)

Supplementary Figure 2. Search keyword for each vaccine type.

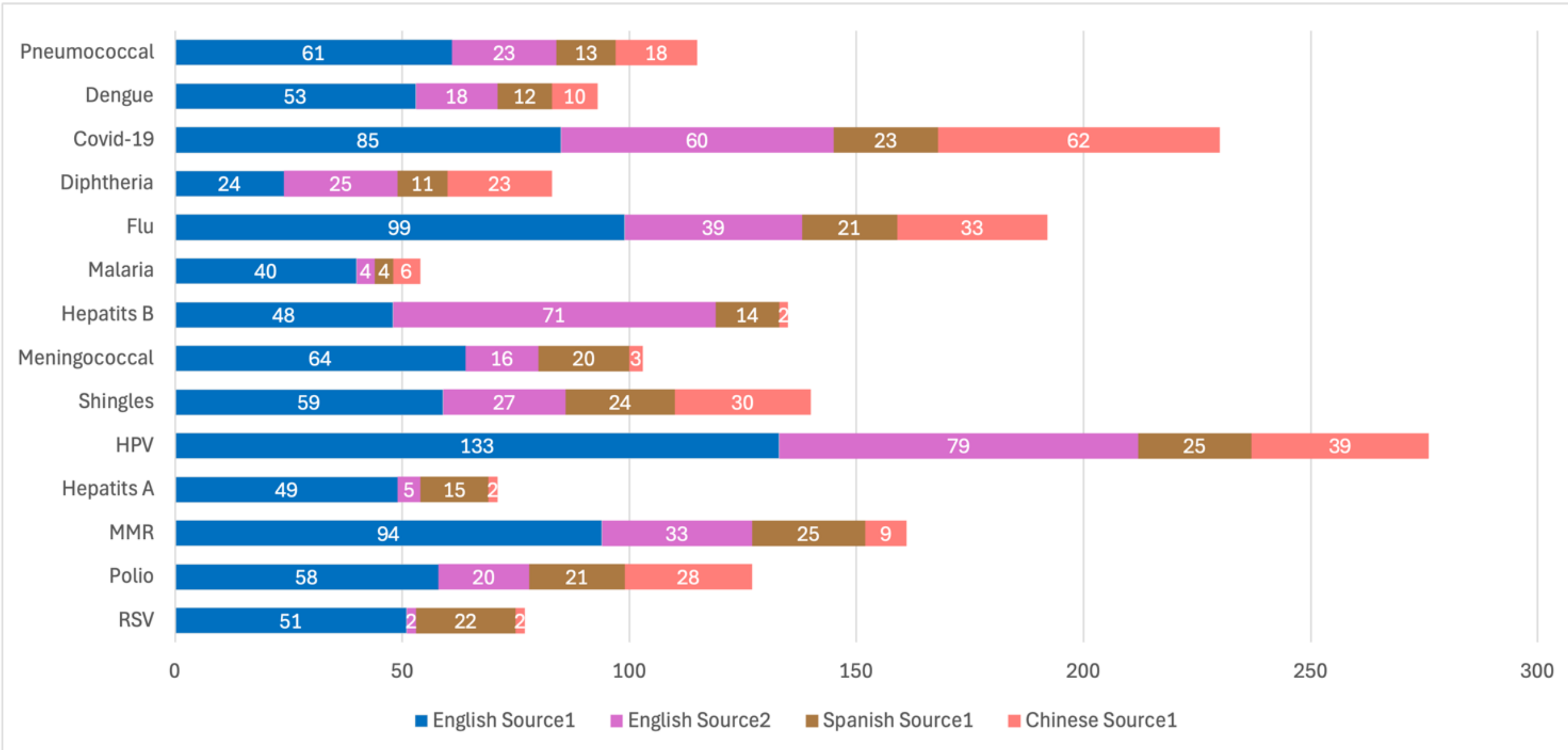

Supplementary Figure 3. Total number of MCQs generated by GPT-4 and extracted from articles for each vaccine type, categorized by language and source.

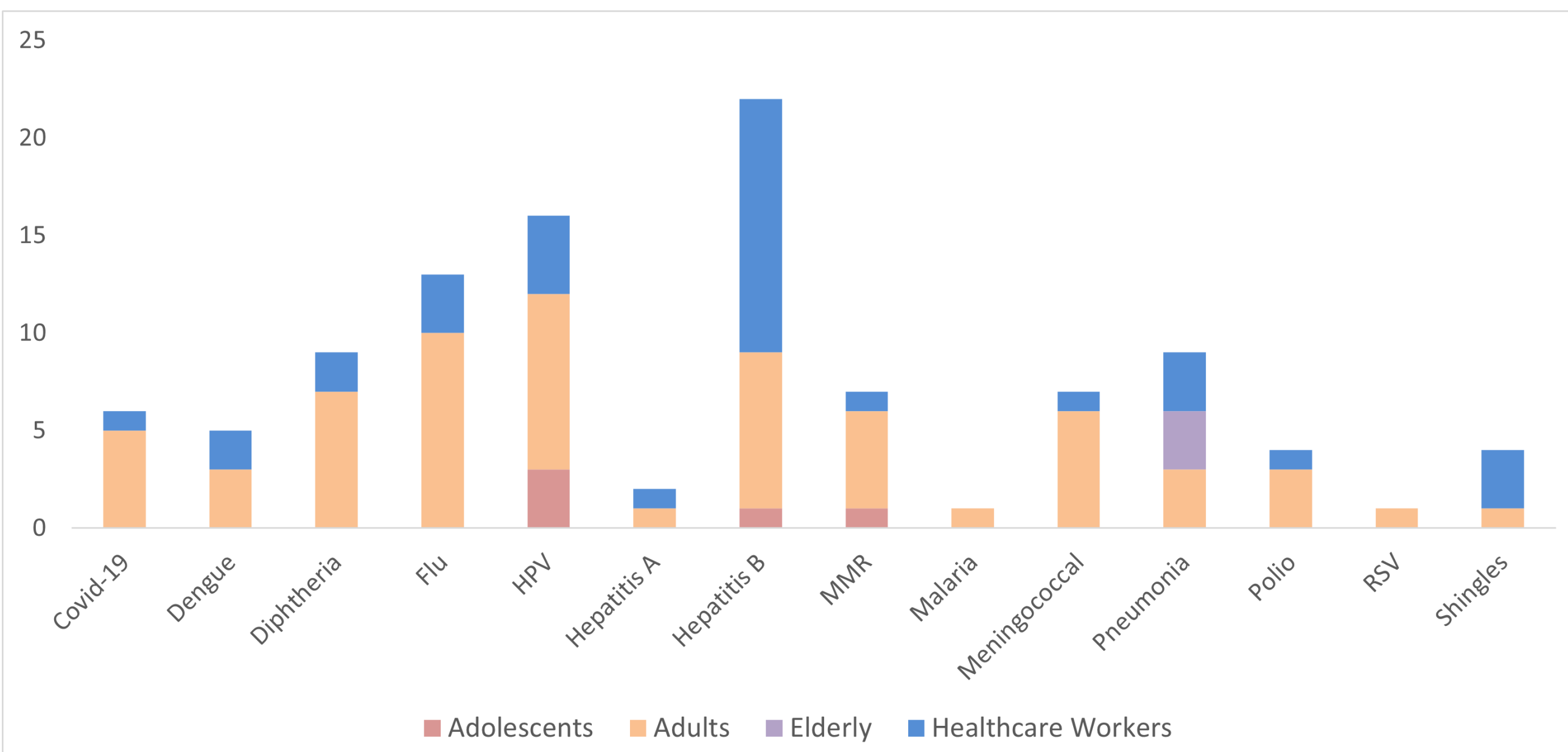

Supplementary Figure 4. Target population distribution by vaccine type in peer-reviewed journals (Source Two).

Chinese	Spanish	English

	Zero shot	Few shot	CoT	Avg	Zero shot	Few shot	CoT	Avg	Zero shot	Few shot	CoT	Avg
Claude 3 Opus	90.20%	87.20%	81.50%		92.00%	92.80%	47.60%		78.30%	88.10%	88.60%	
Gemini 1.5 Pro	36.20%	24.80%	63.80%		76.80%	79.60%	88.40%		89.50%	85.80%	89.80%	
GPT4	82.30%	85.60%	58.60%		93.60%	92.80%	81.20%		86.10%	87.60%	87.00%	
GPT4o	83.00%	88.70%	85.30%		94.40%	93.20%	90.40%	92.7% [a]	90.30%	91.10%	90.00%	90.5% [a]
GPT3.5	79.70%	78.10%	74.30%		84.00%	82.00%	72.80%		74.10%	79.80%	79.00%	
Reka	81.90%	82.30%	82.60%		88.00%	88.40%	87.60%		84.00%	84.90%	81.70%	
01 AI	68.80%	80.40%	73.80%		83.60%	84.00%	70.40%		79.60%	81.60%	79.10%	
Deepseek-v3	89.80%	92.90%	85.30%	89.3% [a]	92.00%	92.40%	90.80%	91.7% [a]	89.10%	90.00%	88.30%	
GLM-4	77.10%	86.10%	84.90%		90.80%	89.20%	54.00%		78.90%	85.10%	82.40%	
GPT-4.5	86.80%	90.60%	86.00%	87.8% [a]	94.80%	94.40%	38.80%		91.30%	88.80%	87.70%	
Grok-3	80.80%	89.80%	43.30%		93.60%	92.40%	58.00%		88.60%	91.40%	90.30%	90.1% [a]
Llama-4	86.00%	89.80%	90.90%	88.9% [a]	95.20%	96.00%	92.00%	94.4% [a]	89.00%	90.40%	89.50%	89.6% [a]
Qwen2.5	83.80%	87.20%	87.20%		94.80%	94.80%	46.00%		86.60%	85.70%	86.60%	
Overall	79.00%	81.80%	76.70%	79.20%	90.30%	90.20%	70.60%	83.70%	85.00%	86.90%	86.10%	86.00%

Supplementary Table 1. Comparison of the overall accuracy among the large language models.
[a] Underlined accuracy values denote the top three performing LLMs for each language on vaccine-related questions.
[b] CoT: Chain of Thought

Variable	Estimate	Std.Error	Z	P.value	OR[a] [95% CI[b]]
Group					
Flagship	0.45	0.03	18.03	0	1.57 [1.50, 1.65]
Vaccine					
Covid-19	-0.17	0.18	-0.98	0.33	0.84 [0.59, 1.19]
Dengue	-1.12	0.23	-4.86	0	0.33 [0.21, 0.51]
Diphtheria	-0.45	0.24	-1.87	0.06	0.63 [0.39, 1.02]
Flu	0.36	0.19	1.88	0.06	1.43 [0.99, 2.06]
Hepatitis A	0.05	0.27	0.17	0.86	1.05 [0.62, 1.76]
Hepatitis B	-0.29	0.19	-1.48	0.14	0.75 [0.51, 1.10]
Malaria	-0.46	0.3	-1.51	0.13	0.63 [0.35, 1.14]
Meningococcal	-0.75	0.23	-3.32	0	0.47 [0.30, 0.74]
MMR	-0.47	0.2	-2.38	0.02	0.63 [0.43, 0.92]
Pneumococcal	-0.84	0.22	-3.82	0	0.43 [0.28, 0.67]

Polio	-0.19	0.21	-0.9	0.37	0.82 [0.54, 1.25]
RSV	-0.74	0.25	-2.94	0	0.48 [0.29, 0.78]
Shingles	-0.71	0.2	-3.52	0	0.49 [0.33, 0.73]
Language					
Chinese	-1.04	0.13	-8.31	0	0.35 [0.28, 0.45]
Spanish	-0.59	0.13	-4.41	0	0.56 [0.43, 0.72]
Method					
Few Shot	0.16	0.03	5.1	0	1.17 [1.10, 1.24]
CoT	-0.23	0.03	-7.84	0	0.79 [0.75, 0.84]
Category					
Prevention[c]	0.68	0.24	2.85	0	1.97 [1.24, 3.13]
Cost[c]	0.01	0.25	0.05	0.96	1.01 [0.62, 1.66]
Effective[c]	0.12	0.14	0.87	0.38	1.13 [0.86, 1.47]
Myths[c]	0.8	0.19	4.22	0	2.23 [1.54, 3.23]
Other[c]	0.66	0.36	1.84	0.07	1.93 [0.96, 3.91]
Regulatory[c]	0.58	0.29	2.02	0.04	1.78 [1.02, 3.13]
Safety[c]	0.22	0.14	1.52	0.13	1.25 [0.94, 1.65]
Types[c]	0.05	0.15	0.32	0.75	1.05 [0.78, 1.42]

Supplementary Table 2. Results of the GLMM: correctness ~ model_group + vaccine_type + language + method + category + (1 | question_id).

[a] OR : Odds Ratio.

[b] CI : Confidence Interval.

[c] Dosing = Dosing & recommendations | Effective = Effectiveness & benefits | Safety = Safety & side effects | Types = Types & basic information | Myths = Misconceptions & corrections | Prevention = Awareness, behavior, & prevention | Cost = Cost and accessibility | Regulatory = Regulatory & monitoring systems | Other

Term	OR[a] [95% CI[b]]	P.value
Source2 vs Source1	0.479 (0.449-0.510)	<0.001

Supplementary Table 3. Mixed-effects logistic regression restricted to English items, examining the association between question source (authority-derived vs questionnaire-derived) and model correctness.

Term	Full Model OR(95% CI)	P.value	Restricted Model[a] OR(95% CI)	P.value

Chinese vs English	0.799 (0.600 - 1.064)	0.124	0.616 (0.456 - 0.832)	0.002
Spanish vs English	0.772 (0.529 - 1.128)	0.181	0.634 (0.427 - 0.943)	0.024
Flagship vs Earlier	1.986 (1.879 - 2.098)	<0.001	1.902 (1.773 - 2.041)	<0.001

Supplementary Table 4 [same Supplementary as GLMM outputs]. Sensitivity analysis comparing full vs restricted model estimates for language and model-group effects.

Model	EN	ES	ZH
GPT-4o	0.87(0.80 – 0.93)	0.88(0.81-0.94)	0.85(0.78-0.92)
DeepSeek-V3	0.91(0.85 – 0.96)	0.89(0.83-0.95)	0.86(0.79-0.92)

Supplementary Table 5. Accuracy on the parallel-language subset across English, Spanish, and Chinese (95% bootstrap confidence intervals).

Panel A: By model

Model	Dosing_Interval	Contraindication	Age_Group	Vaccine_Type	Regulatory_Program	Temporal_Policy	Overgeneralization	Other
GPT-4o	8	4	4	4	1	0	23	6
DeepSeek-V3	4	5	6	4	2	2	24	3
Llama-4-maverick	7	6	4	0	1	0	27	5

Panel B: By language

Language	Dosing_Interval	Contraindication	Age_Group	Vaccine_Type	Regulatory_Program	Temporal_Policy	Overgeneralization	Other
Chinese	3	5	4	0	0	0	10	0
English	13	4	9	6	4	2	55	12
Spanish	3	6	1	2	0	0	9	2

Panel C: By prompt type

Prompt Type	Dosing_Interval	Contraindication	Age_Group	Vaccine_Type	Regulatory_Program	Temporal_Policy	Overgeneralization	Other
CoT	S1	3	1	2	1	2	1	7
	S2	0	1	2	0	0	0	14
Few Shot	S1	4	0	1	1	0	0	6

	S2	2	0	1	0	0	0	4
Zero Shot	S1	4	1	1	3	2	0	12
	S2	0	1	2	1	0	1	12

Supplementary Table 6. Error category distribution of incorrect responses (n = 150), stratified by model backbone, language, and prompting strategy.

Category	Operational Definition	Illustrative Examples
**Dosing and Recommendations**	Questions concerning vaccine dosage schedules, booster timing, number of doses, eligibility criteria, contraindications, or official clinical recommendations.	“How many doses are required?”; “Who should receive the vaccine?”
**Effectiveness and Benefits**	Questions addressing vaccine efficacy, effectiveness, duration of protection, reduction of disease severity, or impact on transmission.	“How effective is the vaccine?”; “How long does protection last?”
**Safety and Side Effects**	Questions related to adverse events, safety concerns, contraindications, precautionary conditions, or clinical safety evidence.	“What are common side effects?”; “Is the vaccine safe during pregnancy?”
**Types and Basic Information**	Questions regarding vaccine composition, mechanism of action, scientific principles, or general background knowledge.	“What does the vaccine contain?”; “How does it work?”
**Regulatory and Monitoring**	Questions concerning regulatory approval status, institutional recommendations, immunization policies, or post-deployment safety monitoring systems.	“What does WHO recommend?”; “How is vaccine effectiveness monitored?”
**Cost and Accessibility**	Questions addressing vaccine pricing, insurance coverage, public funding, supply availability, or access logistics.	“Who covers the cost?”; “What is the projected supply?”
**Misconceptions and Corrections**	Questions reflecting misinformation, myths, unsupported causal claims, or narratives requiring evidence-based correction.	“Vaccines cause infertility.”; “The vaccine caused a public figure’s death.”
**Awareness, Behavior, and Prevention**	Questions concerning preventive rationale, behavioral drivers of vaccination, or public attitudes toward vaccination.	“Why is annual vaccination recommended?”

Category	Operational Definition	Illustrative Examples
**Other**	Assigned only when an item did not clearly align with any predefined category.	—

Supplementary Note 1. Operational definitions and illustrative examples of predefined MCQ content

Error Category	Operational Definition
**Overgeneralization**	Responses that provide vague, overly broad, or nonspecific statements without appropriately applying vaccine-specific clinical constraints required by the question.
**Dosing_Interval**	Errors involving incorrect number of doses, improper spacing between doses, or misinterpretation of primary versus booster schedules.
**Contraindication**	Failure to correctly identify medical contraindications or precautionary conditions (e.g., pregnancy, immunocompromised status) affecting vaccine eligibility.
**Age_Group**	Incorrect application of age-based eligibility criteria, including misinterpretation of approved age ranges or age-specific dosing schedules.
**Vaccine_Type**	Confusion between different vaccine formulations, platforms, or product types (e.g., live attenuated vs. inactivated vaccines), leading to incorrect recommendations.
**Regulatory_Program**	Errors related to misunderstanding formal immunization programs, national schedules, travel requirements, or institutional policy frameworks.
**Temporal_Policy**	Incorrect application of time-sensitive recommendations or outdated guidance, including failure to account for updated policy changes.
**Other**	Errors that do not clearly fit predefined categories, including ambiguous reasoning or multi-factorial mistakes without a dominant identifiable cause.

Supplementary Note 2. Operational definitions of predefined LLM error taxonomy categories
